# Effects of the Pimelic Diphenylamide Histone Deacetylase Inhibitor HDACi 4b on the R6/2 and N171-82Q Mouse Models of Huntington’s Disease

**DOI:** 10.1371/currents.hd.ec3547da1c2a520ba959ee7bf8bdd202

**Published:** 2013-02-05

**Authors:** Jane Y. Chen, Elizabeth A. Wang, Laurie Galvan, My Huynh, Prasad Joshi, Carlos Cepeda, Michael S. Levine

**Affiliations:** Intellectual and Developmental Disabilities Research Center, Semel Institute for Neuroscience and Human Behavior, Brain Research Institute, The David Geffen School of Medicine, University of California, Los Angeles, California; Intellectual and Developmental Disabilities Research Center, Semel Institute for Neuroscience and Human Behavior, Brain Research Institute, The David Geffen School of Medicine, University of California Los Angeles, Los Angeles, CA, USA; Intellectual and Developmental Disabilities Research CenterUCLA; Intellectual and Developmental Disabilities Research Center, Semel Institute for Neuroscience and Human Behavior, Brain Research Institute, The David Geffen School of Medicine, University of California Los Angeles, Los Angeles, CA, USA; Intellectual and Developmental Disabilities Research Center, Semel Institute for Neuroscience and Human Behavior, Brain Research Institute, The David Geffen School of Medicine, University of California Los Angeles, Los Angeles, CA, USA.; Research Professor of Physiology at Intellectual and Developmental Disabilities Research Center, David Geffen School of Medicine, University of California, Los Angeles, CA, USA; Professor at Intellectual and Developmental Disabilities Research Center, David Geffen School of Medicine, University of California Los Angeles, CA, USA, Los Angeles, CA, USA

## Abstract

This report represents a detailed description of experiments designed to replicate and extend the findings of a published study on the effects of treating the R6/2 Huntington’s disease (HD) mouse model with ~300 CAG repeats using the pimelic diphenylamide histone deacetylase (HDAC) inhibitor, HDACi 4b (Thomas et al., 2008). In addition to testing the R6/2 mice, similar experiments examined the effects of the drug on a second transgenic HD mouse model, the N171-82Q mice. As in the original study, the drug was delivered in the drinking water. In the present study we tested larger groups of mice than in the original study. The results indicated that we were unable to replicate the significant behavioral effects of oral HDACi 4b treatment in the R6/2 mice. There were however, non-significant trends for the treated R6/2 mice to be less affected on some of the measures and there were instances of phenotype progression being delayed in these treated mice. In contrast, we did replicate the protection from striatal atrophy in the R6/2 mice. We also did not observe any beneficial effects of HDACi 4b treatment in the N171-82Q mice. Although the behavioral procedures were replicated and an automated activity assessment was added, there were several unexpected complications in terms of solubility of the drug, CAG repeat length differences and gender differences in progression of the phenotype that could have affected outcomes. Clearly more studies will have to be performed using other methods of delivery as well as assessing effects in more slowly progressing HD models to better evaluate the effects of this HDAC inhibitor.

## Notice of Correction

**11 July 2017:** PLOS Currents -. Correction: Effects of the Pimelic Diphenylamide Histone Deacetylase Inhibitor HDACi 4b on the R6/2 and N171-82Q Mouse Models of Huntington’s Disease. PLOS Currents Huntington Disease. 2017 Jul 11 . Edition 1. doi: 10.1371/currents.hd.976177e0cbf724437ea11745c9231a57. View Correction.

## Introduction

A recent study demonstrated therapeutic effects of a new pimelic diphenylamide histone deacetylase (HDAC) inhibitor, HDACi 4b, in the R6/2 mouse model of Huntington’s disease (HD)^1^. The study used the R6/2 strain which expresses exon 1 of the huntingtin (htt) protein with an expanded polyglutamine region of ~300 repeats. This particular strain of R6/2 displays a delayed phenotype compared to the better characterized R6/2 model that has a shorter polyglutamine expansion of ~150 repeats^2^^,^ 3 ^,^ 4 ^,^ 5. The R6/2 mice with 300 repeats exhibit deficits in motor behavior by 12 weeks of age, striatal atrophy, and survive to about 6-7 months. The better characterized R6/2 model (~150 repeats) displays a more aggressive phenotype and mice survive only until 3-4 months and display significant overt behavioral impairments by 40 days of age. Thomas et al. (2008) showed that chronic oral administration of HDACi 4b in drinking water beginning at about 120 days, after the onset of motor deficits, significantly improved motor performance, overall appearance, body weight and striatal atrophy of the R6/2 mice.

In the present report we repeated the procedures for all the behavioral tests as described in Thomas et al., 2008 and we also examined the mice in an automated activity chamber in which both horizontal and vertical movements could be analyzed. We measured body weight separately for males and females which was not done by Thomas et al., 2008. Finally, we measured brain weight as well as striatal and ventricular volumes.

We used two mouse models, R6/2 mice with ~300 CAG repeats and the N171-82Q mice^6^. To replicate the most important portion of the original study we only needed two groups of R6/2 mice, one receiving vehicle treatment in the water and one receiving the HDACi 4b. However, as a precaution, and to make sure that HDACi 4b has no overt deleterious effect on wildtype (WT) mice, we added a group of WTs that received HDACi 4b. The original study was performed with about 8 mice per group. A power analysis using estimates from Figs. 1-3 in Thomas et al. indicated that increasing the sample size to at least 15 mice per group resulted in power estimates of over 0.8 (with α=0.05) on virtually all of the significant measures, even without expected decreases in standard deviation that should occur with larger numbers of animals.

N171-82Q mice begin to show an overt phenotype at about 80 days and progressively become more affected. We began experiments in mice at about 60 days and continued treatment for about 2 months. We tested 4 groups of mice, WT-vehicle, WT-HDACi 4b, N171-82Q-vehicle, N171-82Q-HDACi 4b. Although the N171-82Q mice were not tested in the Thomas et al., 2008 study, more recently this group has published a study demonstrating significant behavioral improvement and prevention of body weight loss after subcutaneous injection of the HDACi 4b^7^ .

## Methods

All procedures were performed in accordance with the U.S. Public Health Service Guide for Care and Use of Laboratory Animals and were approved by the Institutional Animal Care and Use Committee at the University of California, Los Angeles (UCLA). Mice were obtained from R6/2 and N171-82Q breeding colonies maintained at UCLA. Experimental mice were individually housed on a 12h light-dark cycle with lights on 7:00-19:00. Food and water were available *ad libitum*. Genotyping was performed using PCR of DNA obtained from tail samples, once at weaning and again following the completion of behavioral testing to confirm the genotype.

The R6/2 colony (line 4601) was founded initially from mice supplied by The Jackson Laboratory (Bar Harbor, ME) and subsequently maintained by crossing WT C57BL6xCBA-F1 females with hemizygous R6/2 males. CAG repeat lengths for all R6/2 mice were determined by Laragen Inc. (Culver City, CA) and found to be 327±4.4 (n=43). However, due to instability of the CAG repeat length, two groups of R6/2 mice were identified: one with 287±1.9 CAG repeats (n=14) and the other with 347±1.0 CAG repeats (n=29). Because of time and equipment limitations, the R6/2 mice were tested in three cohorts (Table 1.1). A total of 43 R6/2 mice and 20 WT mice were tested. Animals were assigned to the different treatment groups in a semi-randomized way which ensured that there were no significant differences in CAG repeat length or body weight prior to the start of drug treatment. Although there was a slight variation in body weight of R6/2 males at the beginning of experimentation, this difference was not statistically significant.

The N171-82Q colony was established with mice from The Jackson Laboratory (strain 003627) and maintained by crossing WT C57BL6xC3H-F1 females with hemizygous N171-82Q males. Two cohorts of N171-82Q mice were used (Table 1.2). Mice from multiple litters were equally divided by gender and genotype between each treatment group. A total of 42 N171-82Q mice and 48 WT littermates were tested.

**Table 1.1 - Summary of R6/2 cohorts:** 

	R6/2–Vehicle		R6/2–HDACi 4b		WT–HDACi 4b		
	Males	Females	Males	Females	Males	Females	Total
Cohort 1	3	3	3	3	3	3	18
Cohort 2	4	3	4	4	3	3	21
Cohort 3	4	4	4	4	4	4	24
Total	11	10	11	11	10	10	63

**Table 1.2 - Summary of N171 cohorts:** 

	N171-82Q–Vehicle		N171-82Q–HDACi 4bH		WT–Vehicle		WT–HDACi 4bH		
	Males	Females	Males	Females	Males	Females	Males	Females	Total
Cohort 1	8	7	8	6	1	8	2	7	47
Cohort 2	3	3	4	3	7	3	7	3	33
Total	11	10	12	9	8	11	9	10	80

**Table 1.3 - Number of animals for each drug administration group:** 

	Hemizygous–Vehicle		Hemizygous–HDACi 4b(H)		WT–Vehicle		WT–HDACi 4b(H)		
	Males	Females	Males	Females	Males	Females	Males	Females	Total
R6/2	11	10	11	11	--	--	10	10	63
N171-82Q	11	10	12	9	8	11	9	10	80

**Table 2 - Average CAG repeat lengths of R6/2 mice:** 

	# of CAG repeats		
	<300	>300	Average
R6/2 - vehicle	287 ± 3.3 (7)	346 ± 1.9 (14)	327 ± 6.7 (21)
R6/2 – HDACi 4b	287 ± 2.7 (7)	347 ± 0.9 (15)	328 ± 6.2 (22)
Average	287 ± 1.9 (14)	347 ± 1.0 (29)	327 ± 4.4 (43)
WT			(n=20)

**Drug Treatment:**

R6/2 mice and WT littermates were administered 0.85 mg/ml HDACi 4b [N^1^-(2-aminophenyl)-N^7^-phenylheptanediamide] in solution which replaced drinking water for 10 weeks beginning at 17 weeks of age. New drug was given every 3-4 days in clean water bottles and the volume consumed was recorded at each drug change. Control R6/2 mice received an equal amount of hydroxypropyl β-cyclodextrin (HPβCD; MP Biomedicals, Solon, OH) vehicle solution. The concentration of HDACi 4b in solution was monitored daily by UV spectrophotometry. At the start of experimentation, there was not enough HDACi 4b available for the entire experiment. Thus, a second batch of HDACi 4b was purchased and the differences in procedures are detailed below.

***R6/2 Cohort 1. ***HDACi 4b was obtained from Joel M. Gottesfeld at The Scripps Research Institute (La Jolla, CA). For the vehicle solution, HPβCD (24 mg/ml) was dissolved in Arrowhead distilled water pH-adjusted to 5.5 with 1N HCl. To account for moisture in the compound, 0.873 mg/ml of HDACi 4b was added to the vehicle solution, heated to boiling, and cooled in a standing water bath until room temperature.

***R6/2 Cohorts 2 and 3.*** A second lot of HDACi 4b was synthesized by Repligen Corp. (Waltham, MA). The concentrations of HPβCD and HDACi 4b were the same as those used for R6/2 Cohort 1 except for the following procedural changes. Arrowhead distilled water was pH-adjusted to 4.0 with 1N HCl prior to dissolution of HPβCD. After HDACi 4b cooled to room temperature, the solution was filtered with a 0.2µm PES bottle-top funnel filter (Corning).

***N171-82Q Cohorts 1 and 2.*** Due to excessive precipitation of HDACi 4b observed during R6/2 cohort testing, a hydrochloride salt of HDACi 4b was supplied by Repligen Corp. (HDACi 4bH) for use on the N171-82Q cohorts. N171-82Q mice and WT littermates were given HDACi 4bH for 10 weeks beginning at 9 weeks of age. New drug and vehicle were given weekly in clean water bottles and the volume consumed was recorded at each drug change. Because of solvent residue from synthesis of the hydrochloride salt, 1.043 mg/ml of HDACi 4bH was used to reach a target drug concentration of 0.85 mg/ml. HDACi 4bH was added to 50°C HPβCD vehicle solution then heated to and maintained at 95°C until all drug was dissolved. The solution was allowed to cool without a water bath until 40°C, filtered with a 0.2µm PES bottle-top funnel filter (Nalgene) then cooled completely at room temperature. The final pH of the HDACi 4bH solution was recorded and the vehicle solution was pH-adjusted with 1N HCl to match that of the drug solution

**Body Weight and Survival:**

Body weights were recorded at baseline behavioral testing and weekly from the start of drug treatment until euthanasia. All mouse cages were examined daily. Mice were euthanized by intracardial perfusion before the termination of the experiments if they displayed a lack of righting reflex and a lack of movement even after prodding.

**Experimental Procedures:**

Behavioral testing for R6/2 mice was performed at 14 weeks of age for baseline quantification and at 17, 19, 21, 23-27 weeks of age. Baseline for N171-82Q mice was performed at 8 weeks of age and subsequent testing was conducted at 11, 15, and 18 weeks of age. All behavioral testing was performed during the light phase of the light-dark cycle between 8:00 and 12:00. For each week of behavioral testing, all the following measures were assessed in the order listed with open field on the first day, rotarod on the second, and videotaping followed by hindlimb clasping on the third.

***Open Field.*** Open field activity was assessed in a single 12-min session, with 4 data collection bins of 3 min each. Each mouse was placed individually in a square Plexiglas enclosure (40cm X 40cm) equipped with two photobeam sensor rings with a 32 X 32 array of infrared photocells for measuring horizontal and vertical movements (Coulbourn Instruments, Whitehall, PA). Movement was quantified automatically using TruScan software. Data from the entire session and from the first bin of 3 min were analyzed. Outcome measures were distance moved, velocity, stereotypic movement, and number of vertical plane entries. Each stereotypic movement is defined by a series of coordinate changes less than ±0.999 beam spaces (in either the horizontal or vertical plane) and back to the starting point no more than 2 seconds apart. Although stereotypic movements are used to measure repetitive behavior that does not contribute to large location changes, such movements do cause beam breaks and thus contribute to overall distance and vertical plane entry measures. After each session, the equipment was thoroughly cleaned with an ammonium chloride disinfectant (Conflikt; Decon Labs, King of Prussia, PA) to remove residual odors.

***Rotarod. ***Mice were tested on a rotarod (Ugo Basile, Varese, Italy) using a linear accelerating rotation paradigm (5-38 rpm over 10 min). R6/2 mice were trained on the rotarod before the start of drug treatment to establish a behavioral baseline. Baseline training consisted of 3 consecutive days with six trials per day in two sets of three, with 1 min between each trial and at least 3 h between each set. Throughout the duration of the drug treatment, the latency to fall for each mouse was recorded in one set of three trials (1-min intertrial interval). For N171-82Q mice, no baseline training was conducted and the latency to fall for each mouse was recorded in one set of three trials with a 5-min intertrial interval.

The overall latency to fall for each mouse was calculated as the average of all trials performed that week. Occasionally, a mouse fell within 20 sec from the start of the trial. The trial was repeated up to three times. If, on the third try, the mouse still fell in under 20 sec, the last repeat was used to calculate the average for the three trials.

***Hindlimb Clasping. ***Each mouse was assessed for clasping in one set of five trials. For each set, a mouse was suspended by its tail 12 in above an empty cage for 30 sec per trial with a 15 sec intertrial interval. Hindlimb clasping was scored as follows: 0, normal (hindlimbs consistently splayed outward, away from the abdomen); 1, one or two hindlimbs partially retracted toward the abdomen for more than 50% of the time; 2, both hindlimbs fully retracted toward the abdomen for more than 50% of the time; 3, hindlimbs pulled into the body and clasped by the forelimbs.

***Truncal Dystonia (Kyphosis). ***Each mouse was placed in an empty cage and video-recorded for 5 min. Mice were scored for kyphosis as follows: 0, normal; 1, mild kyphosis (not persistent); 2, moderate kyphosis; 3, severe (persistent) kyphosis.

***General Locomotor Activity. ***Each 5-min video recording also was scored for displacement velocity, exploratory behavior, rearing, and grooming as follows: 0, normal; 1, slight reduction (reduced velocity and exploration); 2, moderate reduction (apparent reduction in rearing and grooming in addition to reduced velocity and exploration); 3, severe reduction (mouse stays in place or moves very slowly with no rearing or grooming). Scoring for truncal dystonia and general locomotor activity were done by a researcher blind to treatment and genotype.

**Histology:**

At the end of drug administration and behavioral testing, mice were deeply anesthetized with isoflurane and intracardially perfused with cold 4% paraformaldehyde (PFA) dissolved in PBS. Brains were then removed and postfixed overnight in 4% PFA. Fixed brains were cryoprotected for 2 days in 20% sucrose in PBS then 30% sucrose for 2 days and frozen on dry ice. 30µm slices spanning the striatum from bregma (1.18-0.38mm) were mounted and stained with cresyl violet. Sections 120 µm apart were used in the volumetric analyses. Striatal and lateral ventricular area were manually traced from both sides and calculated by ImageJ Software (National Institutes of Health, Bethesda, MD). Volumes were calculated by multiplying the sum of the areas by the section thickness and recorded in millimeters cubed.

**Statistical Analyses:**

All values in figures and text are presented as mean±SE. Differences among group means were assessed with appropriate ANOVAs (one- or two-way with one repeated measure). Survival data were analyzed with the Kaplan-Meier Mantel-Cox Logrank test. Differences were considered statistically significant if p<0.05. SigmaPlot 12.3 (Systat Software, San Jose, CA) was used to perform all statistical analyses.

## Results

**Survival:**

All R6/2 mice with less than 300 CAG repeats died or were sacrificed before the end of drug treatment. The average age of death was 24.0±0.14 weeks for R6/2-vehicle mice (n=6) and 24.8±0.72 weeks for R6/2-HDACi 4b mice (n=7). There was no significant difference in age of death between treatment groups of R6/2 mice with less than 300 CAG repeats. All R6/2 mice with more than 300 CAG repeats survived until drug treatment ended and were sacrificed for histology. Thus, the survival analysis only includes data from R6/2 mice with less than 300 CAG repeats. Although survival curves for both R6/2 treatment groups were significantly different compared to WTs (p<0.028, Kaplan-Meier Mantel-Cox Logrank test), there was no effect of HDACi 4b treatment on survival of R6/2 mice (Fig. 1A).

Some N171 mice died or were sacrificed before the end of testing. Of mice that died early, the average age of death was 15.3±1.58 weeks for N171-vehicle mice (n=5) and 16.7±1.68 weeks for N171-HDACi 4bH mice (n=3). The log rank statistic for survival curves between treatment groups and genotypes was significantly different (p=0.006; Fig. 1B). However, *post hoc *tests showed that there was only a trend for decreased survival of N171-vehicle compared to WT-vehicle and WT-HDACi 4bH mice (p=0.0839 and p=0.0689, respectively). There were no significant differences in survival between N171-HDACi 4bH and N171-vehicle groups.

**Cumulative survival:**
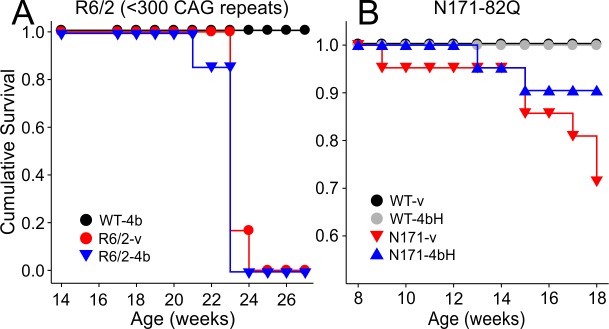
**A**. There were no significant differences in survival between R6/2-vehicle and R6/2-HDACi 4b mice with less than 300 CAG repeats. **B**. Although more N171-vehicle mice died compared to N171-HDACi 4bH mice, the difference in survival was not statistically significant.

**Water Consumption:**

There were no gender differences in water consumption and data for volume consumed by males and females were combined. Averages include data from all R6/2 mice regardless of CAG repeat length. Water consumption for WT littermates of R6/2 mice remained constant at around 5.7±0.12 ml per day throughout the duration of drug administration. There was a significant increase in water consumption with age [F(20,470)=6.264, p<0.001], beginning at 25 weeks for R6/2-HDACi 4b mice and at 26 weeks for R6/2-vehicle mice compared to WTs (p<0.001 and p<0.01, respectively; Fig 2A. Additionally, R6/2-HDACi 4b mice consumed significantly more water than R6/2-vehicle mice at 26 and 27 weeks of age (p<0.02). For this and other measures in order to examine overall effects, data for each mouse that completed the study were averaged over time and then by group. There were no differences in average volume consumed over the length of drug treatment between R6/2-vehicle mice and WTs (p=0.208) or R6/2-HDACi 4b mice (p=0.546) (Fig. 2B). However, the overall average water consumption by R6/2-HDACi 4b mice was significantly higher than that of WT mice (p=0.005) (Fig. 2B). The increase in water consumption probably is a result of the diabetes which occurs in the R6/2 model (Hurlbert et al., 1999).

Water consumption remained constant at approximately 5.5±0.19 and 5.3±0.22 ml per day for WT-vehicle and WT-HDACi 4bH mice, respectively. N171-82Q mice consumed significantly more water than both WT groups [F(30,710)=1.991 p=0.001] from 8-12 weeks of age for N171-vehicle mice and from 8-10 weeks of age for N171-HDACi 4bH mice (Fig. 2C). There were no differences in average volume of water consumed for N171-HDACi 4bH mice compared to WT mice (Fig. 2D). However, N171-vehicle mice consumed significantly more water than WT-vehicle and WT-HDACi 4bH mice (p=0.011 and p=0.003, respectively).

For body weight and the remainder of behavioral analyses, only data from R6/2 mice with more than 300 CAG repeats were used so that all mice were examined over the same time-course and received the same number of tests. Additionally, only data from N171 mice that survived through the end of drug administration were used.

**Water consumption:**
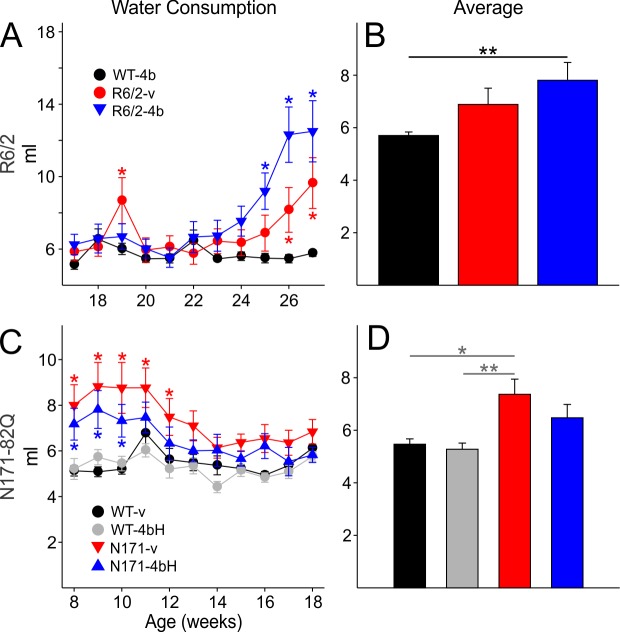
**A**. Water consumption over the duration of drug administration. There was a gradual increase in water consumption for R6/2 mice over time. **B**. Overall, R6/2-HDACi 4b mice consumed significantly more water than WT mice (p<0.01). **C**. Initially, both N171-vehicle and N171-HDACi 4bH groups consumed more water than WTs. However, this was not seen during the later stages of drug administration. **D**. On average, N171-vehicle mice consumed significantly more water than WT-vehicle and WT-HDACi 4bH mice. For A and C, significant differences between mutant and WT mice are indicated by * (p<0.05). For B and D, significant differences in average water consumption are indicated by * (p<0.05) and ** (p<0.01).

**R6/2 Mice**

**Body Weight:**

Previous studies have demonstrated increasing weight loss of R6/2 mice that is more pronounced in males than in females 4 ^,^ 5. Thus, body weight data were separated by gender for analysis. WT males gained weight progressively while R6/2 males displayed a significant decrease in body weight for the duration of treatment [F(22,231)=30.389, p<0.001] (Fig 3A). Percent change in body weight differences between male WT and R6/2-HDACi 4b mice became apparent at 18 weeks of age (p=0.031) while differences between WT and R6/2-vehicle mice began at 19 weeks of age (p=0.012) (Fig 3C). There were no significant changes in weight between treatment groups of R6/2 males. Female R6/2-HDACi 4b mice weighed less than WTs at 24, 25, and 27 weeks of age (p=0.015-0.028) while female R6/2-vehicle mice weighed less only at 27 weeks of age (p=0.021) (Fig. 3B). Female WT mice showed progressive weight gain, as indicated by an increase in percent change of weight over time, while there was little to no weight gain in both groups of female R6/2 mice [F(22,264)=1.717, p=0.026] (Fig. 3D).

**Change in body weight of male and female R6/2 mice:**
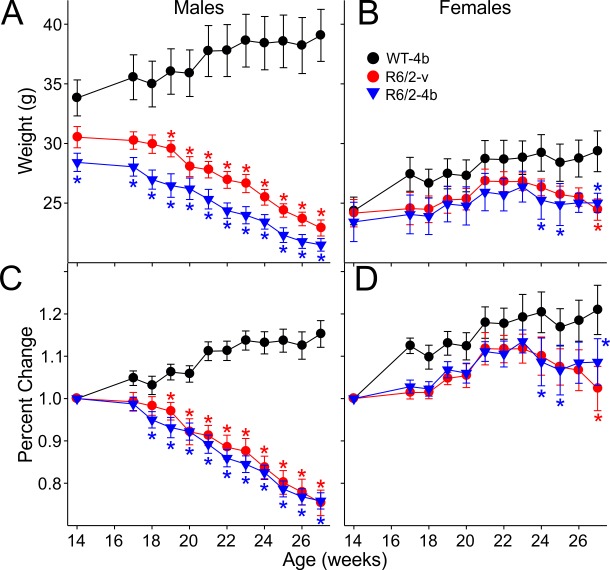
**A, B**. Average body weight of male and female R6/2 mice measured weekly from baseline to the end of drug administration. Male R6/2 mice consistently weighed less than their WT littermates while body weight differences in female R6/2 mice were only seen in the late stages of drug administration. **C, D**. Percent change in body weight of male and female R6/2 mice. Progressive weight loss was observed only in male R6/2 mice. In this figure, significant differences between R6/2 and WT mice are indicated by * (p<0.05).

**Activity-Entire Session:**

There were consistent significant differences in performance of males and females in the open field. Data are first presented for all mice combined for all measures of activity and then separately by gender for measures that showed gender differences. In order to reduce weekly variation in test scores for these analyses, data for each mouse were averaged over time into two epochs, an early epoch from 14-21 weeks and a late epoch from 23-27 weeks. Mice were not tested behaviorally during week 22. For this and other open field measures, the data were analyzed with a two-way ANOVA with one repeated measure (time epoch) followed by Bonferroni *post hoc* tests when main effects and/or interactions were statistically significant. Only the results of the Bonferroni tests will be reported.

In general, both treatment groups of R6/2 mice displayed reduced distances traveled, reduced velocity of movement, increased stereotypies, and reduced rearing. These effects were more prominent during the late epoch. There were no statistically significant differences on any of these measures between the R6/2-vehicle and HDACi 4b groups although on some of the measures in each epoch the HDACi 4b-treated R6/2 mice were slightly less affected. This occurred for measures taken during the entire session as well as measures taken during the first 3 min period (see below).

During the early epoch R6/2-vehicle and drug mice moved less than WTs, but the group differences were not statistically significant (Fig. 4A left group of bars) even though the difference between WTs and R6/2-vehicle mice approached statistical significance (t=2.421 p=0.056). During the late epoch there were significant decreases in total distance between WTs and both groups of R6/2 mice (t=4.21 p<0.001, t=3.102 p=0.009 for comparisons between WTs and R6/2-vehicle and HDACi 4b groups, respectively; Fig. 4A right group of bars).

Velocity of movement was decreased but not significantly in both groups of R6/2 mice compared to WTs in the early epoch. Again, the difference between WTs and the R6/2-vehicle group almost reached statistical significance (t=2.415, p=0.057, Fig. 5B left group of bars). In the late epoch there were significant decreases in movement velocity between WTs and both groups of R6/2 mice (t=4.21 p<0.001, t=3.097 p=0.009 for comparisons between WTs and R6/2-vehicle and HDACi 4b groups, respectively; Fig. 4B right group of bars).

Stereotyped movements increased in both groups of R6/2 mice over the course of testing. In the early epoch, the increase was significant for only the R6/2 vehicle group (t=2.760 p=0.023; Fig. 4C left group of bars). In the late epoch, both groups of R6/2 mice displayed significant increases in stereotypies (t=4.451 p<0.001, t=4.035 p<0.001 for comparisons between WTs and R6/2-vehicle and HDACi 4b groups, respectively; Fig. 4C right group of bars). There was a significant decrease in vertical plane entries in both groups of R6/2 mice but only in the late epoch (t=2.938 p=0.014, t=2.90 p=0.016 for comparisons between WTs and R6/2-vehicle and HDACi 4b groups, respectively; Fig. 4D right group of bars).

**R6/2 activity for the entire open field session:**
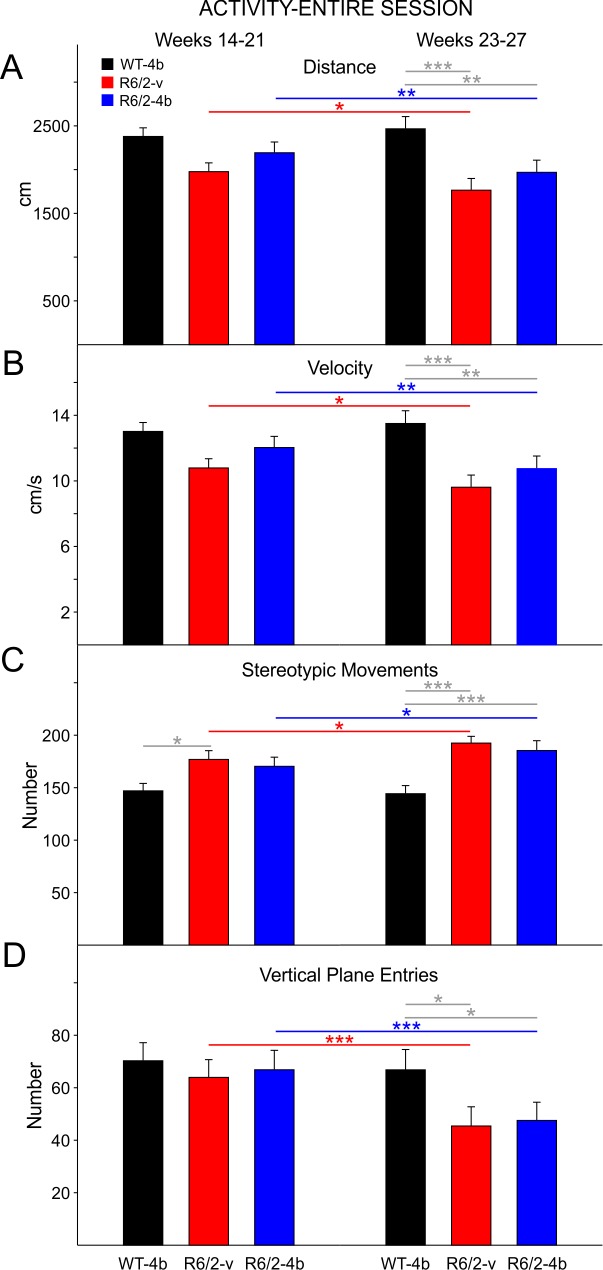
**A**. Total movement distance. **B**. Total velocity of movement. **C**. Total number of stereotypic movements. **D**. Total number of vertical plane entries. In this and other figures, data were divided into an early epoch (14-21 weeks) and a late epoch (23-27 weeks). Both groups of R6/2 mice had decreased movement distance, slower velocity, more stereotypic movements, and fewer vertical plane entries than WTs in the late epoch (right). In this and subsequent figures for R6/2 mice, gray lines highlight differences between R6/2 and WT mice in each time epoch while colored lines show differences between the early and late epochs of each group. Significant differences are indicated by * (p<0.05), ** (p<0.01), and *** (p<0.001).

**Gender Differences in Activity-Entire Session:**

Significant differences in total distance moved occurred only in male mice (Fig. 5A left two panels). For the males, during the early epoch there were no statistically significant differences among the groups although both groups of R6/2 mice moved less than WTs. During the late epoch there were significant decreases in total distance between WTs and both groups of R6/2 mice (t=4.022 p<0.001, t=2.701 p=0.036 for comparisons between WTs and R6/2-vehicle and HDACi 4b groups, respectively; Fig. 5A second panel)

Similarly, velocity of movement significantly decreased only in males in the late epoch (Fig. 5B left two panels). There were significant decreases in movement velocity between WTs and both groups of R6/2 mice (t=4.023 p<0.0001, t=2.959 p=0.037 for comparisons between WTs and R6/2-vehicle and HDACi 4b groups, respectively; Fig. 5B second panel).

Stereotyped movements also increased but only in both groups of male R6/2 mice (Fig. 5C left two panels). In the early epoch, the increase was significant for only the male R6/2 vehicle group (t=2.687 p=0.036). In the late epoch, both groups of male R6/2 mice displayed significant increases in stereotypies (t=3.901 p=0.002, t=3.198 p<0.01 for comparisons between WTs and R6/2-vehicle and HDACi 4b groups, respectively; Fig. 5C second panel). There were no gender differences in vertical plane entries and data are not shown.

**Gender differences in activity of the entire session:**
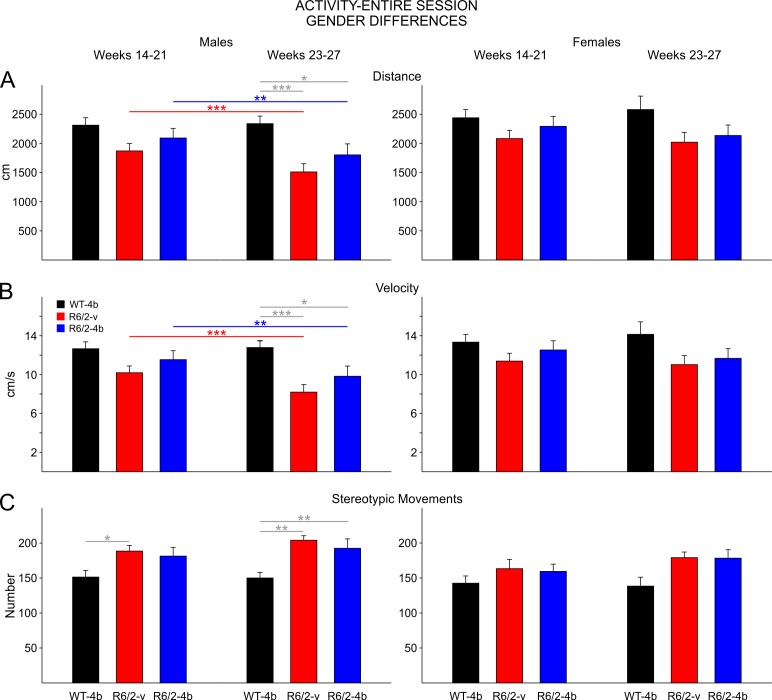
**A**. Total movement distance. **B**. Total velocity of movement. **C**. Total number of stereotypic movements. Significant differences in total activity were only see in male R6/2 mice (left two panels) while there were no differences in activity of female R/62 mice (right two panels). Both groups of R6/2 males had decreased movement distance, slower velocity, and more stereotypies than WT males in the late epoch.

**Activity-First 3min Period:**

The activity data were also analyzed similarly for the first 3 min period to evaluate the initial effects of exposure to the open field. During the early epoch R6/2-vehicle and drug mice moved less than WTs. The difference between the WTs and R6/2-vehicle treated mice was statistically significant (t=2.792 p=0.02, Fig. 6A left group of bars). During the late epoch there were significant decreases in movement distance between WTs and both groups of R6/2 mice (t=5.806 p<0.001, t=4.442 p<0.001 for comparisons between WTs and R6/2-vehicle and HDACi 4b groups, respectively; Fig. 6A right group of bars).

In the early epoch during the first 3 min period, velocity of movement was decreased in both groups of R6/2 mice compared to WTs. The difference between WTs and the R6/2-vehicle group was statistically significant (t=2.665 p=0.029,Fig. 6B left group of bars). In the late epoch there were significant decreases in movement velocity between WTs and both groups of R6/2 mice during the first 3 min period (t=5.518 p<0.001, t=4.102 p=<0.001 for comparisons between WTs and R6/2-vehicle and HDACi 4b groups, respectively; Fig. 6B right group of bars).

Although there were increases in stereotyped movements in both groups of R6/2 mice in the early epoch during the first 3 min period, these were not statistically significant (Fig. 6C left group of bars). During the late epoch, both groups of R6/2 mice displayed significant increases in stereotypies (t=4.229 p<0.001, t=2.827 p=0.018 for comparisons between WTs and R6/2-vehicle and HDACi 4b groups, respectively; Fig. 6C right group of bars). Although there were decreases in vertical plane entries during the first 3 min period in both groups of R6/2 mice, these were only statistically significant in the late epoch (t=4.358 p<0.001, t=3.443 p=0.003 for comparisons between WTs and R6/2-vehicle and HDACi 4b groups, respectively; Fig. 6D right group of bars).

**R6/2 activity during the first 3 minutes of the open field session:**
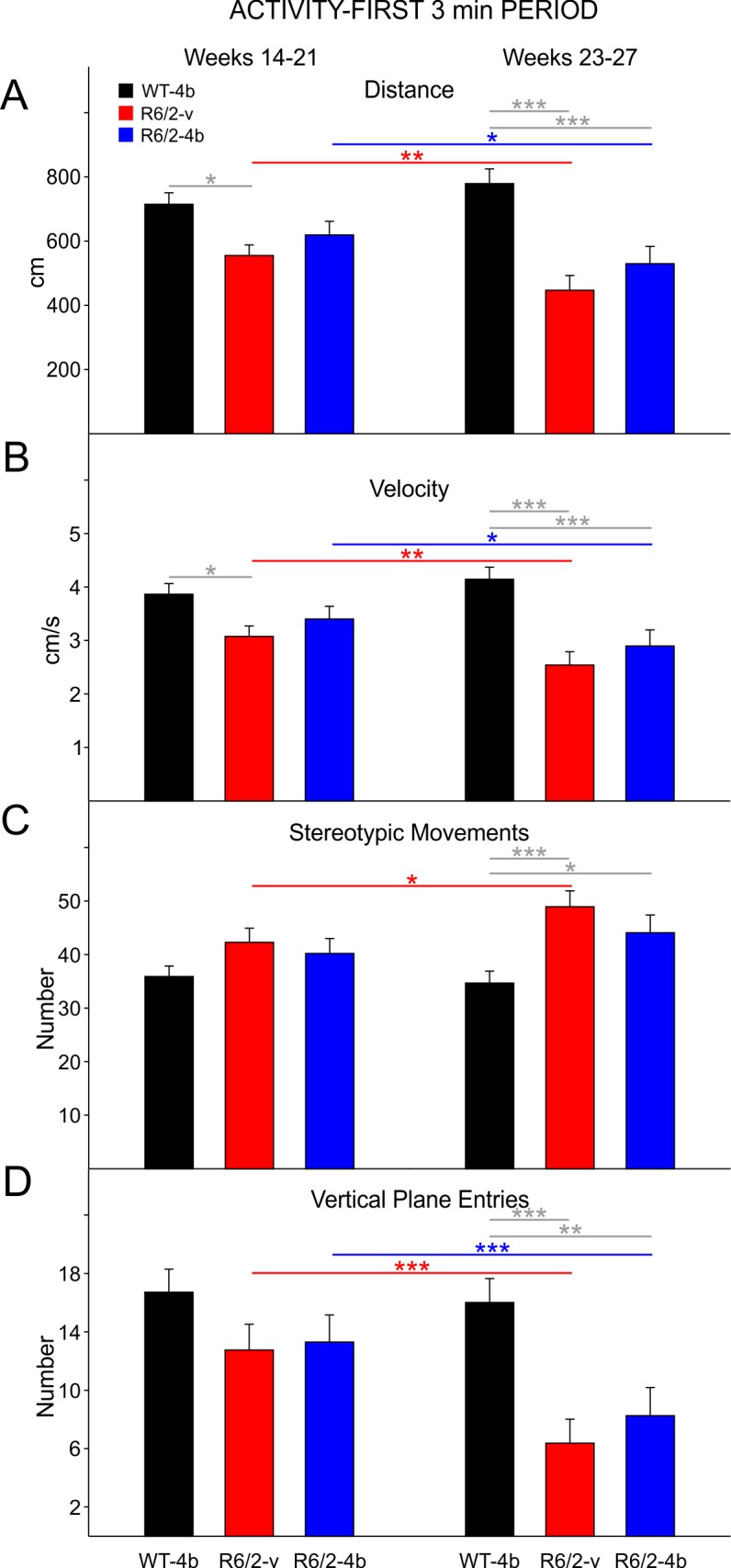
**A**. Movement distance. **B**. Velocity of movement.** C**. Number of stereotypic movements.** D**. Number of vertical plane entries. In the early epoch (left), R6/2-vehicle mice moved less and were slower than WT mice. In the late epoch (right), both R6/2-vehicle and HDACi 4b mice had decreased movement distance, slower velocity, more stereotypic movements, and less vertical plane entries than WT mice.

**Gender Differences in Activity-First 3min Period:**

There were fewer gender differences in activity during the first 3 min period than for the entire session. Interestingly only female R6/2 vehicle mice displayed a significant decrease in total distance during the early epoch (t=2.589 p=0.043; Fig. 7A third panel). Both male and female R6/2 mice moved less than WTs during the late epoch (t=3.980 p<0.001, t=3.119 p=0.011 for comparisons between male WTs and R6/2-vehicle and HDACi 4b groups, respectively; Fig. 7A second panel; t=5.370 p<0.001, t=3.951 p=0.001 for comparisons between female WTs and R6/2-vehicle and HDACi 4b groups, respectively; Fig. 7A last panel).

In females there was a significant decrease in velocity of movement in the R6/2 vehicle group compared to WTs in the early epoch (t=2.522 p=0.05;Fig. 7Bthird panel). Velocity of movement was similarly decreased in both groups of male and female R6/2 mice in the late epoch (t=3.739 p=0.002, t=2.730 p=0.029 for comparisons between male WTs and R6/2-vehicle and HDACi 4b groups, respectively; Fig. 7B second panel; t=5.330 p<0.001, t=3.904 p=0.001 for comparisons between female WTs and R6/2-vehicle and HDACi 4b groups, respectively; Fig. 7B last panel).

Stereotyped movements increased in both males and females in the late epoch (Fig. 7C). The increase in males was significant for both groups of R6/2 mice (t=3.951 p=0.003, t=2.600 p=0.039 for comparisons between male WTs and R6/2-vehicle and HDACi 4b groups, respectively; Fig. 7C second panel) while for females it was only statistically significant for the comparison between WTs and vehicle-treated R6/2 mice (t=2.834 p=0.022; Fig 7C last panel).

Vertical plane entries decreased in both males and females in the late epoch (Fig. 7D). The decrease in males was significant for both groups of R6/2 mice (t=3.396 p=0.005, t=2.762 p=0.027 for comparisons between male WTs and R6/2-vehicle and HDACi 4b groups, respectively; Fig. 7D second panel) while for females it was only statistically significant for the comparison between WTs and vehicle-treated R6/2 mice (t=2.993 p=0.019; Fig 7D last panel).

**Gender differences during the first 3 min of open field:**
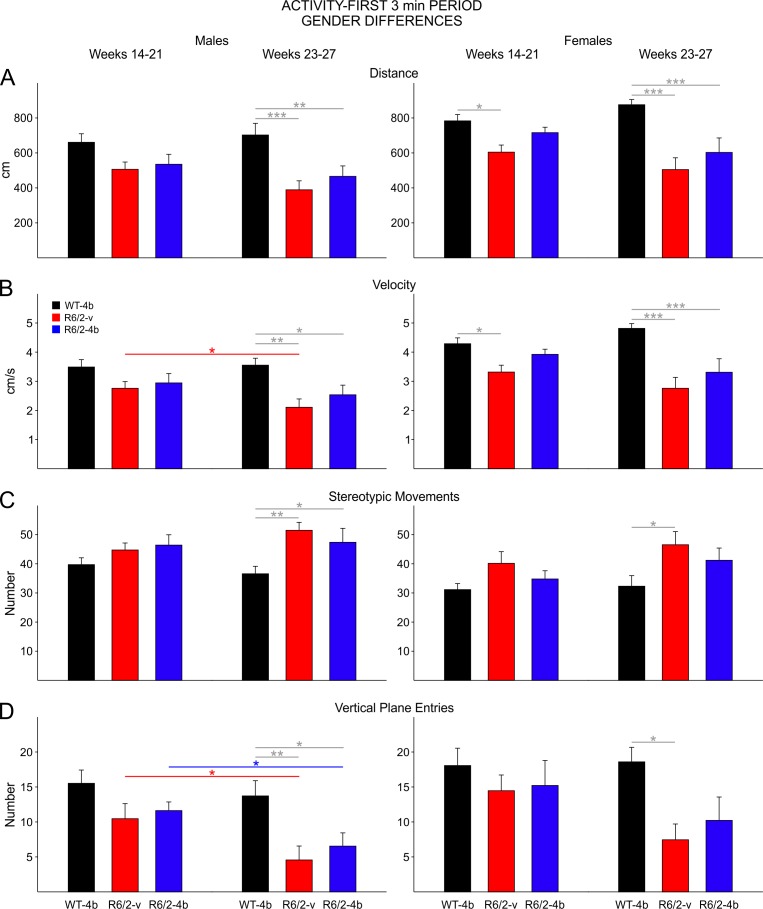
**A**. Movement distance. **B**. Velocity of movement. **C**. Number of stereotypic movements. **D**. Number of vertical plane entries. During the early epoch (first and third group of bars), there was a significant decrease in movement distance and velocity that was only observed in female R6/2-vehicle mice. During the late epoch (second and fourth group of bars), both male and female R6/2-vehicle mice, as well as male R6/2-HDACi 4b mice, showed significant differences in all areas of activity measured. There were no significant differences in stereotypies or vertical plane entries of female R6/2-HDACi 4b mice in the late epoch.

**Rotarod, Clasping, Kyphosis, and Videotaped Open Field:**

The data from all of these measures were analyzed with appropriate two-way ANOVAs similarly to the data from the automated open field activity chamber and divided into an early and late epoch. There were no significant differences in performance of males and females on the rotarod, in clasping or in their kyphosis scores and data were combined.

***Rotarod.***During the early epoch, the two groups of R6/2 mice fell off the rotarod faster than WTs but the differences were not statistically significant (Fig. 8A left group of bars). In the late epoch, although the differences remained, the R6/2 HDACi 4b mice were more impaired and fell significantly sooner than the WTs (t= 2.555 p=0.040, Fig. 8A right group of bars).

***Clasping.*** Although all mice showed some clasping, the R6/2-HDACi 4b treated mice were not as affected in the early epoch as R6/2-vehicle treated mice. Only the increase in clasping score between WTs and the R6/2-vehicle treated mice was statistically significant in the early epoch (t=3.450 p=0.003, Fig 8B left group of bars). There was a trend for the R6/2-HDACi 4b mice to display a decrease in the clasping score compared to R6/2 vehicle-treated mice (t=2.367 p=0.061). In the late epoch only the increase in clasping in the R6/2-vehicle group was significantly different from WTs (t=3.744 p=0.001, Fig. 8B right group of bars). Thus, for this measure the R6/2-HDACi 4b were significantly less affected than R6/2-vehicle treated mice but only in the early epoch indicating a potential delay in development of this particular portion of the phenotype.

**Rotarod performance and clasping score of R6/2 mice:**
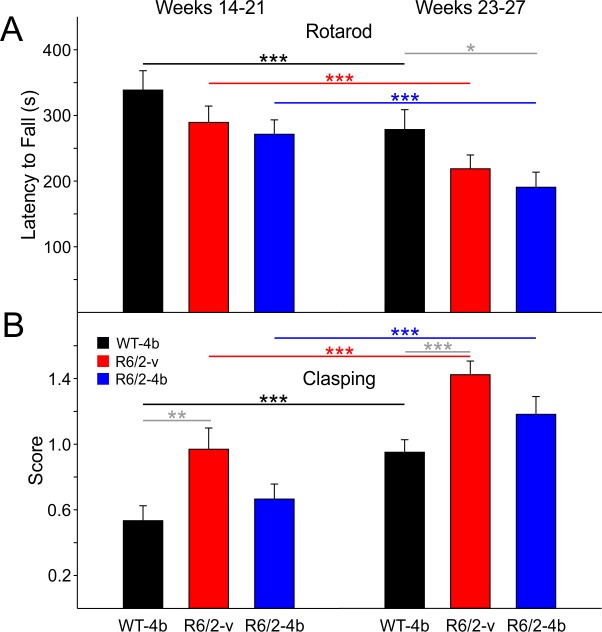
**A**. Average latency to fall from an accelerating rotarod. There were no significant differences in rotarod performance between groups during the early epoch. In the late epoch, R6/2-HDACi 4b mice fell off the rotarod sooner than WT mice. **B**. Average clasping scores. R6/2-vehicle mice had a higher clasping score than WTs during the early epoch that persisted in the late epoch.

***Kyphosis. ***Kyphosis was present in both groups of R6/2 mice in both epochs.****In each epoch the two groups of R6/2 mice displayed significantly higher kyphosis scores than WTs and did not differ from each other (t=5.398 p<0.001, t=5.399 p<0.001 for comparisons between WTs and R6/2-vehicle and HDACi 4b groups, respectively; Fig. 9A left group of bars; t=11.676 p<0.001, t=12.264 p<0.001 for comparisons between WTs and R6/2-vehicle and HDACi 4b groups, respectively; Fig. 9A right group of bars).

***General Locomotor Activity. ***In general, R6/2-HDACi 4b treated mice displayed higher scores indicating that they were more severely affected in the open field ratings. During the early epoch, although both groups of R6/2 mice displayed higher scores only the difference between the R6/2-HDACi 4b group and the WTs was statistically significant (t=2.747 p=0.022, Fig. 9B left group of bars). The increase in score for the R6/2-vehicle group approached statistical significance (t=2.397 p=0.056). In the late epoch R6/2-HDACi 4b mice were the most severely affected and their scores were significantly higher than those of WTs (t=6.130 p<0.001) and the R6/2-vehicle group (t=2.924 p=0.013, Fig. 9B right group of bars). The R6/2-vehicle treated group also displayed a significantly higher score than WTs (t=2.965 p=0.012) in the late epoch.

In the videotaped open field only male R6/2 mice in both groups displayed statistically significant differences during both epochs and the mice that received the HDACi 4b were the most affected in the late epoch (t=2.656 p=0.034, t=3.614 p=0.002 for early epoch comparisons between male WTs and R6/2-vehicle and HDACi 4b groups, respectively; Fig. 9C first panel; t=3.010 p=0.013, t=7.950 p<0.001 for late epoch comparisons between male WTs and R6/2-vehicle and HDACi 4b groups, respectively; t=4.105 p<0.001 for late epoch comparisons between male R6/2-vehicle and HDACi 4b groups, respectively; Fig. 9C second panel).

**R6/2 kyphosis and activity from the videotaped open field:**
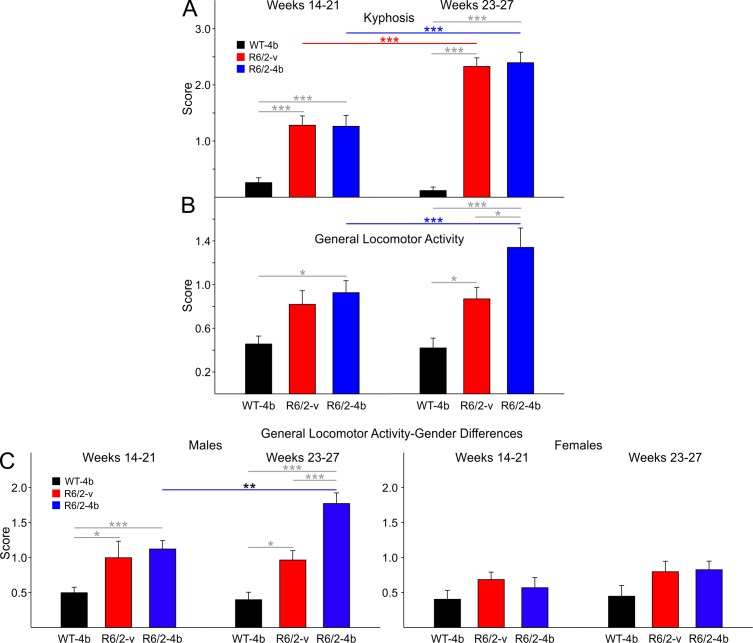
**A**. Average kyphosis scores. Kyphosis was apparent in R6/2-vehicle and HDACi 4b mice during both the early and late epochs. **B**. General locomotor activity scores. R6/2-HDACi 4b mice displayed impaired locomotor activity during the early and late epochs. **C**. Gender differences in general locomotor activity. Significant differences in locomotor activity were seen in male R6/2 mice during both epochs. There were no differences in activity between groups of female mice.

**Histology:**

Brains of R6/2 mice weighed less than those of WTs (0.406±0.009 g for R6/2-vehicle and 0.405±0.010 g for R6/2-HDACi 4b compared to 0.440±0.007 g for WT-vehicle; t=2.835 p=0.019 and t=2.909 p=0.016 for comparisons between WTs and R6/2-vehicle and HDACi 4b, respectively). Striatal atrophy and ventricle enlargement were apparent in brains of R6/2-vehicle mice (Fig 10A compare middle panel with left and right panels). However, this change was not seen in mice treated with HDACi 4b (Fig. 10A right panel). R6/2-vehicle mice displayed statistically significant smaller striatal volumes than WTs and R6/2-HDACi 4b mice (t=2.633 p=0.035 and t=3.090 p=0.011, respectively; Fig 10B). Additionally, the ventricles of R6/2-vehicle mice were larger than those of WT mice (t=3.040 p=0.012; Fig. 10). There were no significant differences in striatal volume or ventricle size between R6/2-HDACi 4b and WT mice.

Since there were significant gender differences in body weight, we also examined gender differences in brain weight, striatal and ventricular volume. When the results were separated by gender, significant differences were not apparent on any of the measures, probably due to decreased numbers of mice per group.

**Changes in striatal and ventricular volumes of R6/2 mice:**
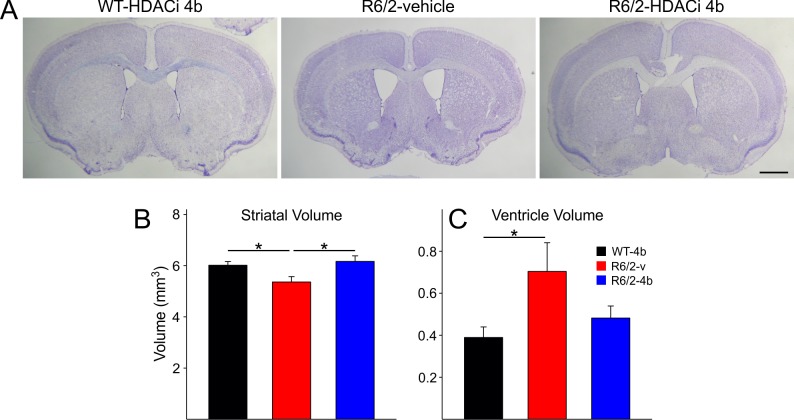
**A**. Representative images of the striatum from WT, R6/2-vehicle, and R6/2-HDACi 4b mice. Scale bar is 1 mm. **B**. Average striatal volumes. R6/2-vehicle mice had smaller striatal volumes than WT and R6/2-HDACi 4b mice. **C**. Average ventricle volumes. R6/2-vehicle mice also had larger ventricles than WTs.

**N171-82Q Mice**

**Body Weight:**

Male N171 mice showed significant differences in body weight compared to WT-vehicle and WT-HDACi 4bH mice beginning at 10 weeks of age for N171-vehicle mice and at 11 weeks of age for N171-HDACi 4bH mice and persisted until the end of drug administration [F(30,320)=31.700 p<0.001] (Fig. 11A). There were no significant differences in weight gain, as indicated by percent change in weight, between WT-vehicle and WT-HDACi 4bH mice or between N171-vehicle and N171-HDACi 4bH mice (Fig. 11C). Female N171-vehicle and N171-HDACi 4bH mice began to show weight differences from WT mice beginning at 13 weeks of age and persisted until the end of drug administration [F(30,350)=13.664 p<0.001] (Fig. 11B). Additionally, female WT-HDACi 4bH mice weighed significantly less than WT-vehicle mice at week 18 (t=2.761 p=0.043). There was an increase of percent change in weight of female WT mice over time, indicating gradual weight gain (Fig. 11D). However, this increase was not observed in female N171 mice and significant differences in weight gain between N171 and WT mice were apparent from weeks 12 through 18 of drug administration [F(30,350)=14.091 p<0.001].

**Changes in body weight of N171-82Q mice:**
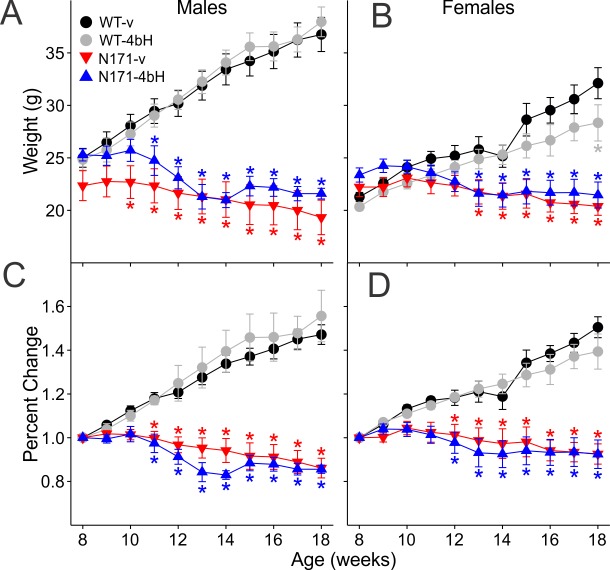
**A, B**. Average body weight. **C, D**. Percent change in body weight over time. Both male and female WT mice gained weight while there was little to no weight change in N171 mice over the duration of drug treatment. Significant differences in body weight between N171 and WT mice are indicated by * (p<0.05).

**Activity-Entire Session:**

Data analyses were performed similarly to those for the R6/2 mice. The early epoch consisted of weeks 8 and 11 and the late epoch weeks 15 and 18 of behavioral testing. Behavioral tests were not conducted during any other weeks. There were no significant differences between genders on the activity measures and data were combined. As with the R6/2 mice, the data were analyzed with two-way ANOVAs with one repeated measure (time epoch) followed by Bonferroni *post hoc* tests. Only the results of the Bonferroni tests are reported.

In general, while the N171 mice differed from WT littermates, especially during the latter epoch of testing, there were no statistically significant differences between N171-vehicle and N171-HDACi 4bH mice on any of the measures.

There were no statistically significant differences in total movement distance, movement velocity, or stereotyped movements between any groups of WT and N171 mice in either the early or late epochs (Fig. 12A-C). During the early epoch, there was a significant decrease in vertical plane entries of N171-HDACi 4bH mice compared to WT-vehicle and WT-HDACi 4bH mice (t=4.227 p<0.001 and t=3.575 p=0.003, respectively; Fig. 12D left group of bars). This reduction in vertical plane entries persisted in the late epoch (t=4.740 p<0.001 and t=3.761 p=0.002 for comparisons between N171-HDACi 4bH and WT-vehicle and WT-HDACi 4bH groups, respectively; Fig. 12D right group of bars). Furthermore, N171-vehicle mice had fewer vertical plane entries than WT-HDACi 4bH mice in the late epoch (t=3.265 p=0.009).

**N171-82Q activity for the entire open field session:**
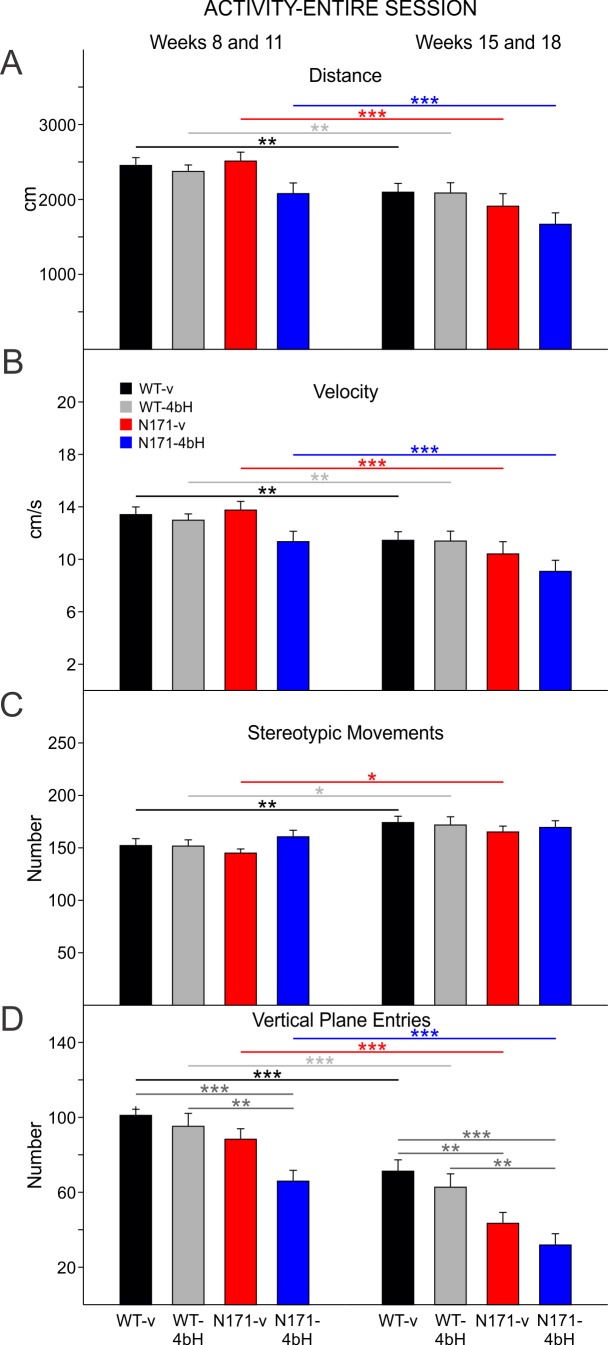
**A**. Total movement distance. **B**. Total velocity of movement. **C**. Total stereotypic movements. **D**. Total vertical plane entries. There were no differences in distance moved, velocity, or stereotypies between N171 and/or WT mice. N171-HDACi 4bH mice had fewer vertical plane entries than WT-vehicle and WT-HDACi 4bH mice during both the early and late epochs. In this and subsequent figures for N171 mice, dark gray lines highlight differences between N171 and WT mice in each time epoch while colored lines show differences between the early and late epochs of each group. Significant differences are indicated by * (p<0.05), ** (p<0.01), and *** (p<0.001).

**Activity-First 3min Period:**

During the early epoch, N171-HDACi 4bH mice moved significantly less than WT-vehicle mice (t=2.736 p=0.043) while there were no significant differences in movement distance among the other groups (Fig. 13A left group of bars). During the late epoch, both groups of N171 mice moved significantly less than WT-vehicle mice (t=3.949 p<0.001 and t=2.852 p=0.031 for comparisons between WT-vehicle and N171-HDACi 4bH and WT-vehicle N171-vehicle mice, respectively; Fig. 13A right group of bars). Additionally, N171-vehicle mice moved less than WT-HDACi 4bH mice in the late epoch (t=3.450 p=0.005).

Decreases in movement velocity of N171-HDACi 4bH mice occurred in the early epoch compared to WT-vehicle mice (t=2.735 p=0.043; Fig 13B left group of bars) as well as in the late epoch when compared to WT-vehicle and HDACi 4bH mice (t=3.927 p<0.001 and t=3.434 p=0.005, respectively; Fig 13B right group of bars). During the late epoch, there also was a decrease in velocity of movement between N171-vehicle and WT-vehicle mice (t=2.866 p=0.030).

There were no differences in stereotypic movements in the first 3 min period of open field between any groups during both the early and late epochs (Fig 13C).

N171-HDACi 4bH mice displayed fewer vertical plane entries compared to WT-vehicle and WT-HDACi 4bH mice during the early epoch (t=4.927 p<0.001 and t=3.244 p=0.009, respectively; Fig 13D left group of bars). This decrease in vertical plane entries of N171-HDACi 4bH mice persisted in the late epoch (*t*=5.845 *p*<0.001 and *t*=4.921 *p*<0.001 for comparisons between N171-HDACi 4bH and WT-vehicle and WT-HDACi 4bH groups, respectively; Fig. 13D right group of bars). Furthermore, during the late epoch, N171-vehicle mice made fewer vertical plane entries than WTs (t=4.724 p<0.001 and t=3.807 p=0.001 for comparisons between N171-vehicle and WT-vehicle and WT-HDACi 4bH groups, respectively).

**N171-82Q activity for the first 3 minutes of the open field session:**
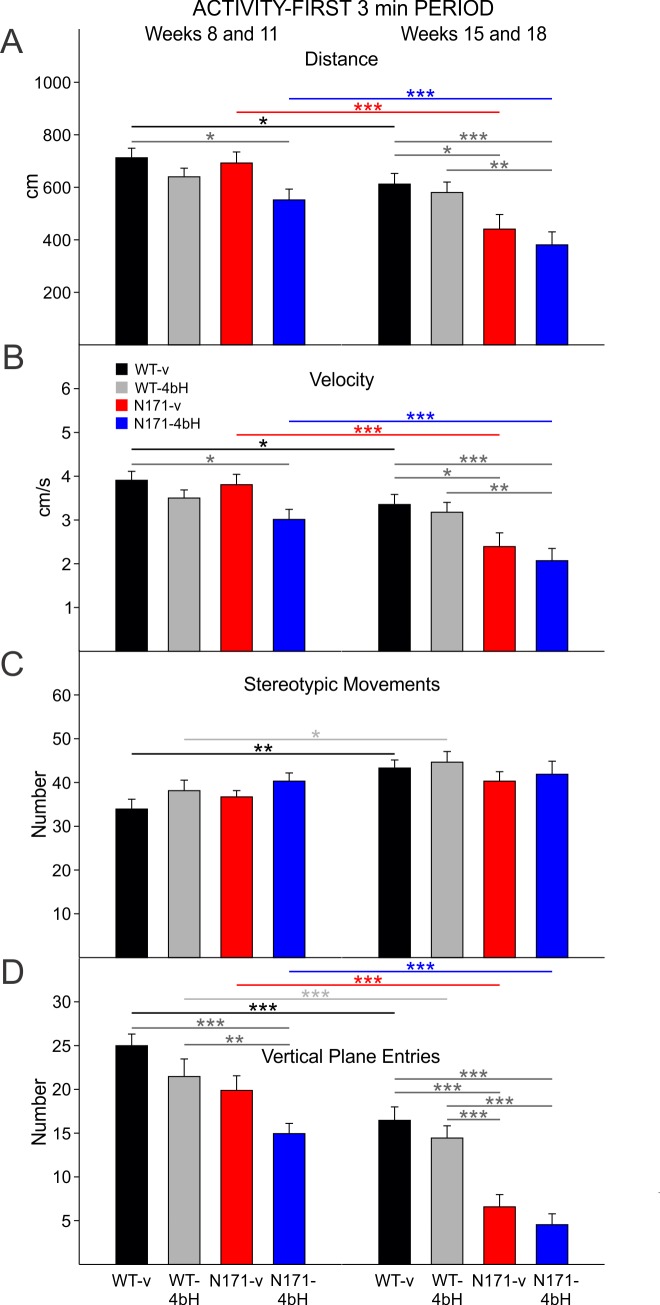
**A**. Movement distance. **B**. Velocity of movement. **C**. Number of stereotypic movements. **D**. Number of vertical plane entries. Overall, N171-HDACi 4bH mice moved less, displayed lower velocity of movement, and fewer vertical plane entries than WTs during the early and late epochs. Similar differences are also seen in N171-vehicle mice in the late epoch.

**Rotarod, Clasping, Kyphosis, and Videotaped Open Field:**

***Rotarod.*** During the early epoch, there was a trend for N171-HDACi 4bH to fall off the rotarod faster than WT-HDACi 4bH mice, but this result did not reach statistical significance (t=2.615 p=0.062, Fig. 14A left group of bars). There were no other differences in rotarod performance between groups in the early epoch. During the late epoch, N171-HDACi 4bH mice fell significantly faster than WT-vehicle and WT-HDACi 4bH mice (t=4.596 p<0.001 and t=3.775 p=0.002, respectively; Fig. 14A right group of bars). Although there was a trend for decreased rotarod performance in the late epoch of N171-vehicle mice compared to WT-vehicle and WT-HDACi 4bH mice, these differences were not statistically significant. These data from the late epoch were re-analyzed separately for males and females since previous reports showed gender differences in rotarod performance (Jia et al., 2012). The analysis indicated that the differences were only significant in males (t=4.464 p<0.001 and t=3.606 p=0.005 for WT-vehicle versus N171-HDACi 4bH and WT-HDACi4bH versus N171-HDACi 4bH; data not shown in the graph).

***Clasping.***Both groups of N171 mice displayed more clasping than WTs during the early and late epochs. In both epochs, the two groups of N171 mice demonstrated significantly higher clasping scores than WTs but did not differ from each other (t=4.036 p<0.001, t=3.948 p<0.001, t=2.848 p=0.032, t=2.743 p=0.043 for comparisons between N171-vehicle and WT-vehicle and WT-HDACi 4bH groups as well as between N171-HDACi 4bH and WT-vehicle and WT-HDACi 4bH groups, respectively; Fig 14B left group of bars; t=5.709 p<0.001, t=5.780 p<0.001, t=6.874 p<0.001, t=6.965 p<0.001 for comparisons between N171-vehicle and WT-vehicle and WT-HDACi 4bH groups as well as between N171-HDACi 4bH and WT-vehicle and WT-HDACi 4bH groups, respectively; Fig 14B right group of bars). Furthermore, N171-vehicle and N171-HDACi 4bH mice displayed an increase in clasping with age, as indicated by a higher clasping score during the late epoch compared to the early epoch (t=3.174 p=0.002 and t=6.849 p<0.001 for N171-vehicle and N171-HDACi 4bH groups, respectively, Fig. 14B).

***Kyphosis. ***There were no significant differences in kyphosis between WT and N171 mice during the early epoch. However, N171 mice displayed a significant increase in kyphosis during the late epoch compared to the early epoch (t=7.129 p<0.001 and t=8.147 p<0.001 for N171-vehicle and N171-HDACi 4bH groups, respectively; Fig. 14C). Furthermore, although the two groups of N171 mice did not significantly differ from each other, N171 mice had significantly higher kyphosis scores than WTs in the late epoch (t=8.333 p<0.001, t=7.230 p<0.001, t=9.512 p<0.001, t=8.379 p<0.001 for comparisons between N171-vehicle and WT-vehicle and WT-HDACi 4bH groups, as well as between N171-HDACi 4bH and WT-vehicle and WT-HDACi 4bH groups, respectively; Fig 14C right group of bars).

***General Locomotor Activity. ***During the early epoch, both groups of N171 mice displayed higher activity scores than WTs (t=3.612 p=0.003, t=2.922 p=0.024, t=3.559 p=0.003, t=2.851 p=0.030 for comparisons between N171-vehicle and WT-vehicle and WT-HDACi 4bH groups, as well as between N171-HDACi 4bH and WT-vehicle and WT-HDACi 4bH groups, respectively; Fig 14D left group of bars). Activity scores for N171 mice were higher in the late epoch than in the early epoch (t=2.712 p=0.008 and t=2.771 p=0.007 for N171-vehicle and N171-HDACi 4bH mice, respectively; Fig 14D). WT-HDACi 4bH mice also showed higher scores during the late epoch when compared to the early epoch (t=3.655 p<0.001). During the late epoch, WT-HDACi 4bH, N171-vehicle, and N171-HDACi 4bH mice demonstrated higher scores than WT-vehicle mice (t=3.147 p=0.012, t=5.289 p<0.001, and t=5.206 p<0.001, respectively; Fig 14D right group of bars).****

**Rotarod, clasping, kyphosis, and general activity of N171-82Q mice:**
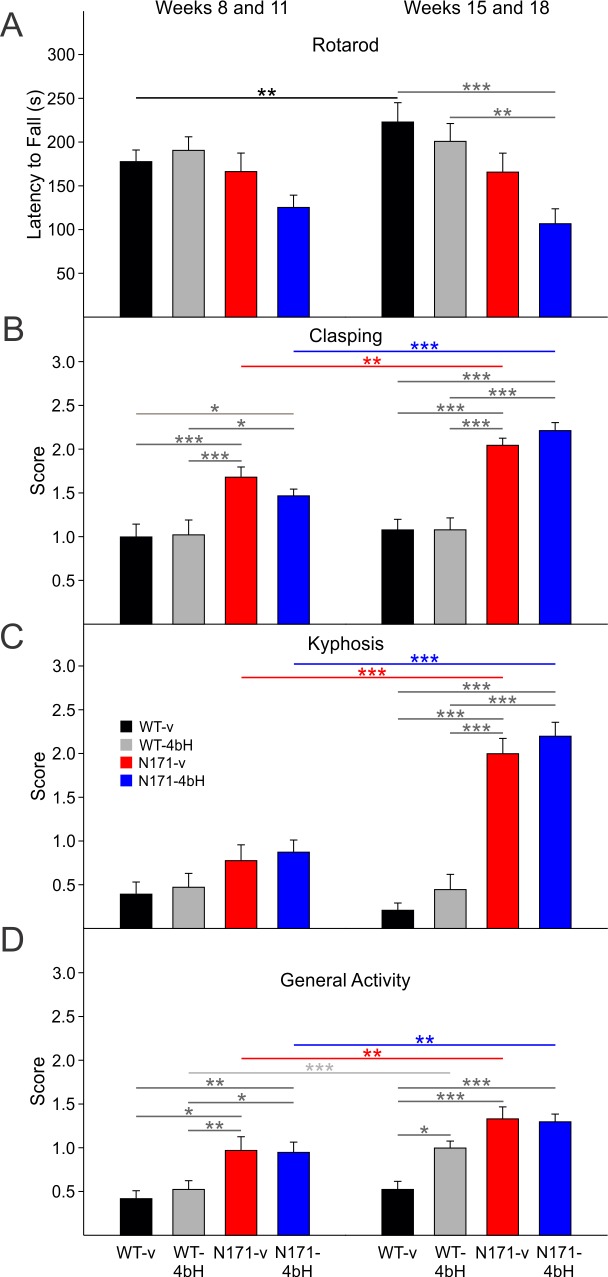
**A**. Latency to fall on an accelerating rotarod. N171-HDACi 4bH mice fell off the rotarod significantly faster than WT-vehicle and WT-HDACi 4bH mice during the late epoch. **B**. Average clasping score. Both groups of N171 mice had higher clasping scores than WT mice throughout the duration of treatment. **C**. Average kyphosis score. Significant kyphosis of N171 mice was observed only in the late epoch. **D**. General locomotor activity. In the early and late epochs, N171 mice displayed decreased locomotor activity compared to WTs. WT-HDACi 4bH mice also showed higher activity scores than WT-vehicle mice in the late epoch. There were no significant differences on any activity measure between N171-vehicle and N171-HDACi 4bH mice.

**Histology:**

Overall, the brains of N171 mice weighed less than those of WT mice (t=4.193 p<0.001 and t=4.068 p<0.001 for comparisons between N171-vehicle and WT-vehicle and WT-HDACi 4bH, respectively; t=5.342 p<0.001 and t=5.225 p<0.001 for comparisons between N171-HDACi 4bH and WT-vehicle and WT-HDACi 4bH, respectively). Average brain weights were: WT-vehicle 0.433±0.009 g, WT-HDACi 4bH 0.431±0.009 g, N171-vehicle 0.374±0.015 g, N171-HDACi 4bH 0.367±0.006 g. Striatal atrophy and ventricle enlargement did not occur in either group of N171 mice.

## Summary and Discussion

The basic finding is that we were unable to replicate the significant behavioral effects of HDACi 4b treatment in the R6/2 mice. There were however, non-significant trends for the treated mice to be less affected on some of the measures and there was one instance of phenotype progression being delayed in the treated mice. In contrast, we did replicate the protection from striatal atrophy in the R6/2 mice. We also did not observe any beneficial effects of HDACi 4b treatment in the N171-82Q mice.

It is interesting that we were able to replicate the prevention of striatal volume reduction and ventricular enlargement in the R6/2 but we did not observe a significant prevention of overall brain weight loss in drug-treated mice. Such a finding suggests that the mechanism of action of the HDACi 4b in the R6/2 mice may be specific for the striatum, at least in the present study. Clearly, such differences should be investigated further, perhaps by examining electrophysiological alterations or morphological changes in the striatum in more detail.

Although we attempted to fully replicate the Thomas et al., 2008 study in terms of the behavioral analyses and the mode of delivery of the HDACi 4b, there were procedural differences that occurred as well as several unexpected complications. The most critical complication was that the HDACi 4b compound, as originally prepared by Thomas et al., 2008 according to their descriptions, did not remain in solution and precipitates formed. Excessive precipitation of the HDACi 4b solution was present even after filtering with a 0.2µm vacuum funnel filter. We used UV spectrophotometry and HPLC analyses to measure solution solubility changes. For the first R6/2 cohort, we did not filter the solution and the amount of HDACi 4b in solution on next day was 61% less than the day the solution was prepared. The solution further degraded to 75% then 82% less on days 2 and 3, respectively. For the next two cohorts the solution was filtered and there was a 55% degradation within 24 h of preparation and subsequently a 73% degradation on days 2 and 3.

As indicated above, due to the excessive precipitation of HDACi 4b observed during R6/2 testing, a hydrochloride salt was supplied by Repligen. The UV spectrophotometry analysis of the HDACi 4bH solution showed no change in concentration over a 7-day period. However, HPLC analysis showed a 50% degradation of HDACi 4bH within 24 h of preparation with 45% benzimidazole present. There was no further change in HDACi 4bH or benzimidazole concentration on subsequent days. The conclusion is that it remains unclear how much of the HDACi 4b each mouse received. Interestingly, toward the end of the experiment the R6/2 mice consumed significantly more water than the WTs probably due to their diabetes^8^. We verified that this increase was not due to spout leakage but was due to increased consumption. Increased water consumption in the N171-82Q mice occurred from 8-12 weeks but not later in the progression of the phenotype as had occurred in the R6/2 mice. As far as we know, diabetes has not been reported in the N171-82Q mice.

A recent publication has questioned the use of oral administration of the HDACi 4b *in vivo* in mouse models^9^. They conclude based on physicochemical properties, metabolic and p-glycoprotein substrate liability that this compound is “…suboptimal to investigate inhibition *in vivo* in mouse models using oral administration.” Although in the present study we did not compare various routes of administration, our findings clearly demonstrate that oral administration in drinking water did not produce robust behavioral effects in either model.

Another difference in the present experiments was the CAG repeat length. As pointed out above our average repeat length was 327±4.4. The repeat length of the Thomas et al., 2008 study was lower, 291-296. Our repeat lengths divided R6/2 mice into two groups, a smaller group with 287±1.9 and a larger group with 347±1.0 repeats. All R6/2 mice with less than 300 CAG repeats died by 25 weeks of age during the study while the others survived until the end of drug administration. It is certainly feasible that the group with smaller repeat lengths in the present study was already too severely affected by phenotype progression to demonstrate benefit from the drug treatment. Similarly, it is possible to argue that the mice with the longer repeat length did not show a severe enough phenotype to display benefit. If HDACi 4b only works in such a small therapeutic window of CAG repeats, its benefit for HD patients using an oral administration route of administration also would become difficult to evaluate.
