# Correction: Effects of the Pimelic Diphenylamide Histone Deacetylase Inhibitor HDACi 4b on the R6/2 and N171-82Q Mouse Models of Huntington’s Disease

**DOI:** 10.1371/currents.hd.976177e0cbf724437ea11745c9231a57

**Published:** 2017-07-11

**Authors:** PLOS Currents

## Correction

There is an error in the second author’s name. The correct name is Elizabeth A. Wang. The correct citation is Chen JY, Wang EA, Galvan L, Huynh M, Joshi P, Cepeda C, Levine MS. Effects of the Pimelic Diphenylamide Histone Deacetylase Inhibitor HDACi 4b on the R6/2 and N171-82Q Mouse Models of Huntington’s Disease. PLOS Currents Huntington Disease. 2013 Feb 5 . Edition 1. doi: 10.1371/currents.hd.ec3547da1c2a520ba959ee7bf8bdd202.

## Reference

Chen JY, Wang E, Galvan L, Huynh M, Joshi P, Cepeda C, Levine MS. Effects of the Pimelic Diphenylamide Histone Deacetylase Inhibitor HDACi 4b on the R6/2 and N171-82Q Mouse Models of Huntington’s Disease. PLOS Currents Huntington Disease. 2013 Feb 5 . Edition 1. doi: 10.1371/currents.hd.ec3547da1c2a520ba959ee7bf8bdd202.

